# Factors associated with time to first birth interval among ever married Bangladeshi women: A comparative analysis on Cox-PH model and parametric models

**DOI:** 10.1371/journal.pgph.0004062

**Published:** 2024-12-18

**Authors:** Sarmistha Paul Setu, Rasel Kabir, Md. Akhtarul Islam, Sharlene Alauddin, Mst. Tanmin Nahar

**Affiliations:** 1 Statistics Discipline, Science Engineering and Technology School, Khulna University, Khulna, Bangladesh; 2 School of Public Health Sciences, University of Waterloo, Waterloo, Ontario, Canada; The University of Newcastle Australia: University of Newcastle, AUSTRALIA

## Abstract

The fertility rate of a married woman can be measured by the length of the first birth interval (FBI). This length is influenced by some significant factors. Better knowledge about the factors affecting the birth interval can help in controlling population growth and fertility progress. The main focus of this study was to compare the performance of Cox-Proportional Hazard (Cox-PH) and the parametric Accelerated Failure Time (AFT) model in assessing the impact of significant factors affecting the time to FBI of ever-married Bangladeshi women. Information of 14941 women having at least one birth was included in this study from the most recent nationally representative data 2017–18 Bangladesh Demographic and Health Survey (BDHS). We used the Cox-PH model and AFT model under various parametric forms of survival time distributions (Weibull, Exponential, and Log-normal distribution) to measure the effect of factors influencing FBI. And then, a respective Akaike information criterion (AIC) was calculated for selecting the best-fitted model. According to the AIC and BIC values, the log-normal model fitted better than other AFT models. Based on the log-normal model, women’s age and age at first marriage, maternal and paternal education, contraceptive use status, used anything to avoid pregnancy, sex of household head, and spousal age difference had a significant association with FBI of ever married Bangladeshi women. The parametric AFT model (log-normal distribution) was a better fitted model in evaluating the covariates associated with FBI of ever-married Bangladeshi Women. Higher education, the right age at marriage, and proper knowledge about family planning (i.e., contraception use) should be ensured for every married person to control the gap of the first birth.

## Introduction

The time between two successive live births is referred to as the birth interval. And the time between marriage to first birth is known as the first birth interval (FBI). The mean age where the woman conceives their first kid especially is of great interest to researchers and the general public. A woman’s first childbirth symbolizes the beginning of her parenting journey. It already has a strong association with fertility, which influences every woman’s future survival. The time of life under which a woman starts reproducing has to control the number of babies during herbirth through the entire reproductive phase or reproductive longevity. As a result, the maternal age at first birth affects the size and fertility rate that might have had in the whole lifetime, which also impacts the population’s size, diversity, and growth prospects [1]. Marriage to FBI is an actual incidence in the life of women with increasing responsibilities. The FBI has an impact on the length of successive birth intervals and affects the reproductive pattern of women [2]. Premature delivery, low birth weight, small gestation period, births, and perinatal mortality are almost all connected to short intervals [3, 4]. Examining birth intervals provides insight into birth spacing patterns and maternal, infant, and childhood mortality [5]. Short birth intervals are characterized as those under two years and have adverse effects on maternal, perinatal, and neonatal outcomes and child health [6]. However, the precise mechanisms are poorly understood [7].

On the contrary, short birth intervals are associated with an increased risk of death for the mother and children. Studies have shown that children born less than 24 months after a previous sibling also risk poor health [8]. Short birth intervals also threaten maternal health. According to the Bangladesh Demographic and Health Survey 2017–18 report, birth intervals are generally long in Bangladesh, with a median interval of 55.7 months [9].

Prolonged breastfeeding and a long period of postpartum amenorrhea are likely to contribute to the relatively high percentage of births occurring after 24 months or more in Bangladesh [10]. More than half percent of non-first births occur within three or more years after the previous birth [9, 11], while only a slight percent of births occur 24–35 months after the previous birth [9, 12]. Eleven percent of children are born after an interval considered’ too short,’ i.e., less than 24 months [13]. A comparison with earlier Bangladesh demographic health surveys shows that the median birth interval in Bangladesh has increased significantly over time, rising from 35 months in 1993–1994 to 44 months in 2007, 47 months in 2011, 52 months in 2014, and 56 months in 2017–18, marking an approximate 56 percent increase between 1993 and 2017–18 [9]. The waiting marriage to first birth signifies a couple’s fertility at an earlier marriage stage. As the first birth is the most welcome event for almost all families in Bangladesh, there is a slight chance of memory lapse in reporting the date of first marriage and the date of first birth [14]. Women at first birth in developing countries significantly affect the population’s demographic characteristics. Thus, women’s age at practical marriage and age at first childbirth are proximate determinants of fertility behaviour [15]. Marriage to the FBI was significantly different for the age of women at marriage, region, education of women, and marriage cohort in Ethiopia. Previous research has indicated that woman’s education level, age, place of living, job position, and use of contraceptives all impact the age at which she delivers childbirth in Indonesia [16]. It should be noted that education level has a considerable impact on women’s marital decisions. Furthermore, lower-income women are more likely to begin parenthood earlier, to have more kids and face greater difficulties during birth [16, 17]. Another study found that the spouse’s age and occupation difference had not affected the wedding to the FBI [18]. The determinants of FBI in rural Bangladesh, respondent’s age, age of women at marriage, family income, and quality of care at the clinic were significant determinants investigated by a previous study [19]. Religious status has a substantial link with the time to first birth, which is consistent with previous research findings [20]. After a disastrous outcome of the last delivery, maternal and neonatal mortality is also higher, which is mediated mainly through short birth intervals [21, 22]. Birth to pregnancy intervals of 18 months had the most significant neonatal and newborn death rates.

Months are slightly increased from birth to pregnancy at 18–27 months [23]. The most updated WHO recommendation for a successful pregnancy is at least two years, translating to a delivery delay of 33 months when nine months of gestation is used [24, 25]. The Bangladeshi guidelines suggest a three-year gap between the previous live birth and the next attempt at conception, equivalent to a 45-month birth-to-birth interval assuming a nine-month gestation period [24, 26]. Over the last 30 years, there has been an increase in the usage of contraception (from 25.3% in 1986 to 52% in 2017–18) [9, 27]. Bangladesh has witnessed significant reductions in fertility and marked increases in the median birth interval, increasing from 35 months in 1993–1994 to 47 months in 2011 [24]. However, in Bangladesh, more than two-thirds of women marry before 18, and the typical age at first birth is around 18 years [26]. The birth intervals for these young women are only 27 months, and there are considerable disparities between socio-economic categories, with impoverished, less schooled women having short periods [28]. The prior sibling’s survival is strongly linked to the length of the birth gap [7, 22, 29]. Children whose prior sibling died had a median birth interval 15 months shorter than children whose previous sibling is living, according to the Bangladesh Demographic and Health Survey 2017–18 (29 and 58 months, respectively) [9]. In women, even without a diagnosis of polycystic ovarian syndrome, a higher BMI at age 18 was a predictor of ovulatory infertility. Lower Body mass index at age 18, considered to be obese, is a risk factor for subsequent ovulatory infertility [30]. Mother’s age at first birth, parity, previous birth interval, mother’s working status, gender composition of the living children, and mass media are also described as determinants of birth intervals [14, 28, 31].

There are several studies that used only one method of Cox-PH and AFT models [32–34]. Accelerated Failure Time (AFT) models can simply regresses the logarithm of the survival time over the covariates [35]. And some researches [33, 34] showed AFT model as a good alternative to the Cox regression model in the analysis of survival observations. So far, there is no study using Bangladesh Demographic and Health Survey 2017–18 data that checks PH assumptions of Cox Proportional Hazard model and exhibits the difference of using Cox and AFT model for determining the significant factors affecting the timing of the first birth interval (FBI) [36].

The major goal of this study was to examine if the semi-parametric (Cox-PH) outperforms parametric AFT models in determining the impact of some key variables on the FBI of ever-married Bangladeshi women and find the best-fitted model to identify the influential factors affecting the timing of first birth interval (FBI).

## Methods

### Study design and data sources

The dataset used to conduct the analysis of this study was taken from 2017-18BDHS. The dataset included the characteristics of women aged 15–49 years in Bangladesh. It was directed by the National Institute of Population Research and Training (NIPORT) of the Ministry of Health and Family Welfare (MOHFW), and a Bangladeshi Research Firm named Mitra and Associates accomplished the survey. The United States Agency for International Development (USAID) provided all financial support needed to run the survey. The dataset is available on the website of the DHS program [37]. An enumeration area (EA) with an average of about 120 households was considered as a primary sampling unit (PSU) for conducting the survey. For the sake of collecting data for this study, they used a two-stage stratified cluster sampling method. They selected 675 EAs (250 EAs in urban areas and 425 in rural areas) with a probability proportional to the size of EA, in the first stage of sampling. Then they made a complete list of households in all chosen EAs for making a sampling frame for the second stage. In the second stage, a systematic sample of 30 households was selected from the sampling frame.

In this study, we included only the first birth history of each woman. The FBI is the length of time between the date of marriage and the date of first live birth. Here, the lengths of time were calculated by subtracting the date of birth of the first child from the date of first marriage. We only dealt with ever-married women of Bangladesh who met their first live birth. BDHS 2017–18 contained data of 20127 ever-married women. Among them, 18134 women met their first childbirth. We excluded women whose birth intervals were less than eight months (cmc). Because minimum, 37 weeks (8 months) is required to give birth a normal child [38]. After excluding sterile women (women who didn’t give birth within 15 years after their marriages were considered as primary sterile), negative birth interval, and missing information, finally, we included about 14941 women to assess the significant factors affecting the FBI of ever-married women in Bangladesh.

#### Response variable

In this study, the birth interval was the response variable, which can be defined as the length of time between marriage to first birth measured in months.

#### Explanatory variables

The explanatory variables which might determine the change in FBI of women were socio-economic, demographic, health, and environmental factors. Several predictors were considered in the earlier studies to investigate the essential relative contribution of different potential risk factors to the change in FBI. We included here women’s age at first marriage, current age, residence, division, religion, mother’s and partner’s education, mother’s occupation, contraceptive using status, wealth index, spousal age differences, ever used anything to avoid getting pregnant, and sex of household as a significant covariates which were found to be associated with the FBI of women [13, 15, 32, 39–45].

#### Statistical analysis

As this study included a censored dataset, different methods of survival analysis were carried out. Initially, a Log-rank test was conducted to compare the survival between different groups. A popular semi-parametric model (Cox-PH Model) was used to estimate the association between the FBI and other relevant socio-economic and demographic factors. Moreover, parametric survival models with different baselines (Exponential distribution, Log-normal distribution, and Weibull distribution) were also examined. We used the Cox-PH model and three commonly used AFT models (Exponential distribution, Log-normal distribution, and Weibull distribution) which were used in the earlier studies to determine the factors affecting the FBI of ever-married women in Bangladesh [13, 32, 46, 47]. The estimation of the Cox-PH model and three parametric AFT models (Exponential distribution, Log-normal distribution, and Weibull distribution) were performed by using R-studio version 4.0.5 with packages ‘Survival’ and ‘flexsurv’ respectively.

#### Cox Proportional Hazard model

Let *Y*_*i*_ denote the observed time (either censoring time or event time) for subject *i*. Let *C*_*i*_ be the indicator that the time corresponds to an event (i.e. if *C*_*i*_ = 1 the event occurred and if *C*_*i*_ = 0 the time is a censoring time). Let *Xi* = {*X*_*i*1,_ …, *X*_*ip*_} be the realized values of the covariates for subject i. The hazard function for the Cox proportional hazard model has the form-

h(tXi)=h0(t)exp(β1Xi1+β2Xi2+…+βpXip)=h0(t)exp(Xi.β)

This expression gives the hazard rate at time t for subject i with covariate vector (explanatory variables) *Xi*. = (*x*_1_, *x*_2_,…, *x*_*p*_)′ is the values of the vector of explanatory variables for a particular individual, and *β* = (*β*_1_, *β*_2_,…, *β*_*p*_) is a vector of regression coefficients. The corresponding survival functions are related as follows:

S(tX)=s0(t)exp(∑βixi)

This model, also known as the Cox regression model, makes no assumptions about the form of *h*_0_(*t*) (Baseline Hazard) [48].

#### Parametric Accelerated Failure Time model

*Exponential distribution*. The exponential model is the most simplest of all life distribution models with only one parameter [49]. The exponential model has got a constant hazard rate. The probability that an individual will die within a small interval of time Δ*t* given that the person has survived up to time t is constant for any time period. The probability density function of an exponential distribution is given as:

$$f\left(t\right)=\lambda e^{-\lambda t};t>0\;and\;\lambda >0$$

The cumulative distribution function denoted as *F*(*t*), the probability that an individual has survived up to time t is given as:

$$F\left(t\right)=1-e^{-\lambda t},0\leq t<\infty$$

The survival function *S*(*t*), the probability that an individual survives beyond time t can be derived from *F*(*t*) as follows:

$$S\left(t\right)=P\left(t\right)=1-F\left(t\right)=e^{-\lambda t}$$

The hazard function denoted as *h*(*t*) is given as:

$$h\left(t\right)=\frac{f(t)}{S(t)}=\lambda$$

*Log-normal distribution*. According to the Log-normal distribution is a very flexible distribution just like the Weibull distribution, therefore it can fit many types of failure data [50]. The two-parameter Log-normal distribution with scale parameter *σ* and shape parameter (median time) has a probability density function given as below:

$$f\left(t\right)=\frac{1}{\sigma t\sqrt{2\pi }}exp\left(-\frac{1}{2\sigma^{2}}{\left[lnt-ln\tau_{m}\right]}^{2}\right);0<t<\infty$$

The survival function is given by:

$$S\left(t\right)=1-F\left(t\right)=1-\phi \left[\frac{lnt-ln\tau_{m}}{\sigma }\right]$$

$$F\left(t\right)=\int_{0}^{t}\frac{1}{\sigma t\sqrt{2\pi }}exp\left(-\frac{1}{2\sigma^{2}}\left[lnt-ln\tau_{m}\right]\right)dx=\phi \left[\frac{lnt-ln\tau_{m}}{\sigma }\right]$$

The hazard rate denoted as h(t) is derived as follows:

$$h\left(t\right)=\frac{f(t)}{S(t)}=\frac{\frac{1}{\sigma t\sqrt{2\pi }}exp\left(-\frac{1}{2\sigma^{2}}{\left[lnt-ln\tau_{m}\right]}^{2}\right)}{1-\phi \left[\frac{lnt-ln\tau_{m}}{\sigma }\right]}$$

*Weibull distribution*. The exponential distribution is not applicable in some of lifetime data like biological and social process because it assumes a constant hazard rate. The Weibull distribution is the most flexible and more general distribution, it has got two parameters; one of the parameters is called the scale parameter and the other the shape parameter [51].

The probability density function of the Weibull distribution is formulated in a number of similar forms, but one of the commonly used versions is given by:

$$f\left(t\right)=\frac{\alpha }{t}{\left[\frac{t}{\lambda }\right]}^{\alpha }e^{-\frac{t}{\lambda }},0<t<\infty$$

Where, *λ and α* are the scale and shape parameters respectively. The Weibull distribution is a more general distribution compared to the exponential distribution, because when *λ* = 1, the Weibull distribution simplifies to an exponential distribution. The cumulative density function denoted as *F*(*t*) is given as:

$$F\left(t\right)=1-e^{-{\left(\frac{t}{\alpha }\right)}^{\lambda }}$$

The survival function *S*(*t*) is given as:

$$S\left(t\right)=e^{-{\left(\frac{t}{\alpha }\right)}^{\lambda }}$$

It follows that the hazard rate is given by:

$$h\left(t\right)=\frac{\alpha }{t}{\left(\frac{t}{\lambda }\right)}^{\left(\alpha -1\right)}$$

The Weibull model can fit a range of survival data because of its flexibility. When *α* = 1 the shape parameter is set to one, the failure rate is a constant. When *α* ≥ 1, the failure rate is increasing and when *α* < 1, we have a decreasing failure rate.

*AIC*. To compare different parametric models, we used Akaike Information Criterion (AIC). It is measure of the goodness of fit of an estimated model. The value AIC was calculated in the following way:

AIC=-2Loglikelihood+2p

Where, p is the number of parameters in the model (model specific and covariates) [52].

*BIC*. To compare different parametric models, we used Bayesian Information Criterion (BIC). The value BIC was calculated in the following way:

BIC=-2Loglikelihood+logk*p

Where k is the number of observations and p is the number of parameters in the model (model specific and covariates).

The estimation of the Cox-PH model and three parametric AFT models (Exponential distribution, Log-normal distribution, and Weibull distribution) were performed by using R-studio version 4.0.5 with packages ‘Survival’ and ‘flexsurv’ respectively [53].

*Ethics statement*. We have used a secondary dataset from the Demographic and Health Surveys (DHS) Program website (https://dhsprogram.com/data/). No ethics approval is required for this dataset.

## Results

There were 14941 respondents for the analysis of this study. Out of the total respondents of this study, 13257 (88.73%) women had at least one child, and the rest, 1684 (11.27%) were censored (women without a child until the end of the interview). The average FBI was 47.57 months with a standard error of 0.52 months and a median of 26 months. The women’s average age was 31.22 ± 0.08 years, and the age at first marriage was 16.19 ± 0.02 years.

Here, Table 1 represents the frequency of some demographic characteristics and their KM estimates (mean ± S.E) for women’s FBI (months) with their respective p-values of the log-rank test. The results of Table 1 showed that women’s age at first marriage, current age, religion, occupation, education, status of avoiding pregnancy, contraceptive using status, partner’s education, wealth index, spousal age difference, residence, and division had a significant impact on FBI at 1% level of significance. This suggests that the probability of giving first birth after marriage was significantly different in the group of women whose marital age was less than 18 years from mothers whose marital age was greater than or equal to 18 years and higher for mothers whose marital age was greater than or equal to 18 years. Women who were illiterate had a significantly different probability of giving first birth compared to women having primary, secondary and higher education, and the survival probability was higher for women having primary, secondary and higher education groups. Women aged less than 21 years old had a higher probability of delaying their first birth. Similarly, the probability of having a first birth was higher in women who used contraception than women who did’t use contraception. There was a significance difference in the probability of giving first birth among women from different divisions. Women with high wealth index and lower spousal age differences had a higher probability of delaying their first pregnancy.

**Table 1:** Demographic characteristics of women and their Kaplan-Meier estimates for the FBI (months).

Covariate	No (%)	FBI (Months)		Log Rank Test p-value
		Mean ± SE	Median	
**Age at first marriage(years)**				
<18 Years	10982(73.50%)	78.75±1.31	28	<0.001
≥18 Years	3959(26.50%)	39.81±1.02	24	
**Religion**				
Muslim	13492(90.30%)	69.15±1.07	27	<0.001
Non-Muslim	1449(9.70%)	57.41±2.70	24	
**Mother’s education**				
Illiterate	2106(14.10%)	46.03±1.36	29	<0.001
Primary	4352(29.10%)	48.99±1.32	25	
Secondary and Higher	8483(56.80%)	85.02±1.59	27	
**Partner’s education**				
Illiterate	3133(21.0%)	106.41±2.94	32	<0.001
Primary	4670(31.30%)	71.05±1.85	27	
Secondary and Higher	7138(47.80%)	49.77±1.06	25	
**Residence**				
Urban	5530(37.0%)	71.10±1.70	28	0.019
Rural	9411(63.0%)	66.39±1.24	26	
**Mother’s occupation**				
Not-Working	7598(50.90%)	81.86±1.63	27	<0.001
Working	7343(49.10%)	54.73±1.16	26	
**Mother’s age(Years)**				
≤ 20 Years	2096(14.0%)	213.27±4.62	43	<0.001
21 to 30 Years	5416(36.20%)	61.95±1.61	24	
>30 Years	7429(49.70%)	39.43±0.55	28	
**Ever used anything to avoid getting pregnant**				
No	1565(10.50%)	199.25±5.25	84	<0.001
Yes	13376(89.50%)	53.97±0.87	25	
**Contraceptive use status**				
No	5714(38.20%)	107.57±2.18	32	<0.001
Yes	9227(61.80%)	45.02±0.85	24	
**Sex of household head**				
Male	13189(88.30%)	68.20±1.08	27	0.150
Female	1752(11.70%)	68.99±2.92	28	
**Division**				
Barisal	1655(11.10%)	64.26±2.79	28	<0.001
Chittagong	2060(13.80%)	63.67±2.58	23	
Dhaka	2277(15.20%)	75.54±2.77	28	
Khulna	2026(13.60%)	70.61±2.69	30	
Mymensingh	1646(11.0%)	67.04±2.96	28	
Rajshahi	1930(12.90%)	67.59±2.69	29	
Rangpur	1858(12.40%)	63.39±2.67	26	
Sylhet	1489(10.0%)	61.98±2.93	22	
**Spousal age difference (Years)**				
≤ 5 year	4835(32.40%)	74.60±1.87	29	<0.001
6 to 10 year	5962(39.90%)	68.11±1.60	26	
≥11 year	4144(27.70%)	60.60±1.74	25	
**Wealth Index**				
Poor	5476(36.70%)	60.85±1.48	26	<0.001
Middle	2876(19.20%)	65.56±2.22	26	
Rich	6589(44.10%)	74.86±1.63	28	

From Table 2, it can be seen that among 13 covariates, PH assumption meet only for the 4 covariates (religion, residence, sex of household head, and Spousal age difference (Years)) at 5% level of significance. That is most of the covariates didn’t meet the proportional hazard assumption. The overall global test also suggested that there was an indication (P-Value = 0.000) of non-proportional hazards over time.

**Table 2:** Test the proportional hazard assumptions of Cox regression.

Covariates	*χ*^2^value	Df	P-Value
Age at first marriage (years)	19.84	1	<0.001
Religion	3.37	1	0.067
Mother’s education	181.03	2	<0.001
Partner’s education	18.90	2	<0.001
Residence	0.33	1	0.566
Mother’s occupation	93.83	1	<0.001
Mother’s age (Years)	1075.11	2	<0.001
Ever used anything to avoid getting pregnant	76.50	1	<0.001
Contraceptive use status	60.03	1	<0.001
Sex of household head	0.36	1	0.54
Division	151.91	7	<0.001
Spousal age difference (Years)	1.87	2	0.394
Wealth Index	9.57	2	0.008
Global Test	1319.27	24	<0.001

**Key note:** ^Df^ Degree of Freedom

As the PH assumption was violated for the data of this study, we then represented the parameter estimation of three commonly used AFT models (Weibull distribution, exponential distribution, and Log-normal distribution) along with CoxPH in Table 3 for identifying the impact of each parameter on the survival time (FBI). Here we used three AFT models (Weibull distribution, exponential distribution, and Log-normal distribution) because the AFT model did’t have any PH assumption.

**Table 3:** Results of fitting the Cox Proportional Hazard model (PH) and parametric Accelerated Failure Time (AFT) model.

Covariates	PH model		Weibull AFT model		Exponential AFT model			Log-normal AFT model	
	$e^{\left(\hat{\beta }\right)}$	p-value	$\hat{\beta }$	p-value	$\hat{\beta }$	p-value		$\hat{\beta }$	p-value
Intercept			6.746	0.000	6.657	0.000		5.512	<0.001
**Age at first marriage (years)**									
<18 Years^Ref^	1.00								
≥18 Years	1.19	<0.001	-0.27	<0.001	-0.26	<0.001	-0.25		<0.001
**Religion**									
Muslim^Ref^	1.00								
Non-Muslim	1.04	0.133	-0.03	0.321	-0.03	0.263	-0.03		0.399
**Mother’s education**									
Illiterate^Ref^	1.00								
Primary	1.13	<0.001	-0.16	<0.001	-0.16	<0.001	-0.15		<0.001
Secondary and above	1.07	<0.001	-0.07	0.020	-0.07	0.007	-0.09		<0.001
**Partner’s education**									
Illiterate^Ref^	1.00								
Primary	1.19	<0.001	-0.26	<0.001	-0.24	<0.001	-0.24		<0.001
Secondary and above	1.31	<0.001	-0.39	<0.001	-0.37	<0.001	-0.37		<0.001
**Residence**									
Urban^Ref^	1.00								
Rural	1.03	0.083	-0.05	0.024	-0.05	0.009	-0.04		0.101
**Mother’s occupation**									
Not-working^Ref^	1.00								
Working	1.01	0.342	-0.03	0.145	-0.03	0.073	-0.01		0.621
**Mother’s age (Years)**									
≤ 20 Years^Ref^	1.00								
21 to 30 Years	2.46	<0.001	-1.35	<0.001	-1.29	<0.001	-0.89		<0.001
>30 Years	2.36	<0.001	-1.42	<0.001	-1.38	<0.001	-0.83		<0.001
**Ever used anything to avoid getting pregnant**									
No^Ref^	1.00								
Yes	2.43	<0.001	-1.22	<0.001	-1.15	<0.001	-1.02		<0.001
**Contraceptive use status**									
No^Ref^	1.00								
Yes	1.25	<0.001	-0.34	<0.001	-0.34	<0.001	-0.27		<0.001
**Sex of household head**									
Male^Ref^	1.00								
Female	1.05	0.053	-0.10	<0.001	-0.09	<0.001	-0.01		<0.001
**Division**									
Barisal^Ref^	1.00								
Chittagong	1.36	<0.001	-0.33	<0.001	-0.30	<0.001	-0.47		<0.001
Dhaka	1.02	0.449	0.01	0.724	0.02	0.440	-0.07		0.103
Khulna	0.95	0.203	0.07	0.056	0.08	0.021	0.02		0.531
Mymensingh	1.01	0.840	0.00	0.946	0.01	0.806	-0.05		0.262
Rajshahi	0.99	0.994	0.02	0.604	0.02	0.423	-0.03		0.424
Rangpur	1.06	0.105	-0.03	0.458	-0.01	0.631	-0.12		0.004
Sylhet	1.30	<0.001	-0.27	<0.001	-0.243	<0.001	-0.47		<0.001
**Spousal age difference (Years)**									
≤ 5 (years) ^Ref^	1.00								
6 to 10(years)	1.13	<0.001	-0.17	<0.001	-0.17	<0.001	-0.16		<0.001
≥11 years	1.20	<0.001	-0.25	<0.001	-0.24	<0.001	-0.25		<0.001
**Wealth Index**									
Poor^Ref^	1.00								
Middle	0.98	0.500	0.02	0.339	0.03	0.233	0.01		0.645
Rich	0.89	<0.001	0.16	<0.001	0.15	<0.001	0.12		<0.001

**Key note:** ^Ref^Reference, ^P-Value^Probability Value, ^AFT^Accelerated Failure Time, ^PH^Proportional Hazard

From Table 3, using the Cox PH model, women’s age at first marriage, current age, education, status of avoiding pregnancy, contraceptive use status, partner’s education, spousal age difference covariates are statistically significant at a 1% level of significance, and the covariate sex of household head is significant at 5% level of significance. But the covariates of religion, women’s occupation, residence, division, and wealth index exhibit an insignificant relationship with the FBI.

Moreover, in the case of Weibull, exponential and Log-normal distribution, women’s current age, age at first marriage, education, partner’s education, status of avoiding pregnancy, contraceptive use status, sex of household head, spouse age difference showed significant association with FBI at 1% level of significance. But covariate residence is significantly associated with FBI for only Weibull, with an exponential distribution at a 5% level of significance. But in the case of Log-normal distribution, the type of place of residence is insignificant.

We calculated the AIC, BIC, and log-likelihood values of the three parametric in Table 4. AFT models, Weibull, Exponential, and Log-normal distributions to determine the best-fitted model. The three parametric models, Weibull, Exponential, and Log-normal distribution, had AIC values of 138066.90, 139693.80, and 135791.80, respectively. And their respective BIC values are 138878.0, 139996.7, and 138451.6. And Log-normal distribution had the lowest Log-likelihood value of -67893.92. That means the Log-normal distribution had the lowest Log-likelihood, AIC, and BIC value among the three models (three parametric AFT models). The Log-normal distribution was the best-fitted model for FBI of Bangladeshi women.

**Table 4:** Comparison of three parametric AFT models.

Model	Log-Likelihood	AIC	BIC
Weibull	-69031.44	138066.90	138878.0
Exponential	-69845.92	139693.80	139996.7
Log normal	-67893.92	135791.80	138451.6

**Key note:** ^AIC^Akaike Information Criterion, ^BIC^Bayesian Information Criterion

Finally, we calculated the estimates of hazard ratio with the respective p-value and 95% confidence interval of Log-normal distribution for each covariate associated with FBI. This is represented in Table 5. Age at first marriage (years) showed a significant positive relationship with first birth of women at a 1% level of significance. Here, the hazard ratio of less than 1 indicates a longer duration between marriage to first birth, and a hazard ratio of greater than 1 indicates the shorter duration between marriage to the child’s first birth.

**Table 5:** Estimates of hazard ratio (95% CI) and P-value of log-normal distribution for FBI according to different categorical variables.

Variable	HR (95% CI)	p-value
**Age at first marriage (years)**		
<18 Years^Ref^	1.00	
≥18 Years	1.19 (1.15–1.24)	<0.001
**Religion**		
Muslim^Ref^	1.00	
Non-Muslim	1.05 (0.99–1.11)	0.133
**Respondent’s education**		
Illiterate^Ref^	1.00	
Primary education	1.13 (1.07–1.19)	0.000
Secondary and above	1.07 (1.01–1.14)	0.010
**Partner’s education**		
Illiterate^Ref^	1.00	
Primary education	1.19 (1.14–1.26)	<0.001
Secondary and above	1.31 (1.25–1.37)	<0.001
**Residence**		
Urban^Ref^	1.00	
Rural	1.03 (0.99–1.07)	0.082
**Respondent’s occupation**		
Not working^Ref^	1.00	
Working	1.01 (0.98–1.05)	0.342
**Respondent’s age(Years)**		
≤ 20 Years^Ref^	1.00	
21 to 30 Years	2.46 (2.29–2.63)	<0.001
>30 Years	2.36 (2.20–2.53)	<0.001
**Ever used anything to avoid getting pregnant**		
No^Ref^	1.00	
Yes	2.43 (2.25–2.62)	<0.001
**Contraceptive use status**		
No^Ref^	1.00	
Yes	1.25 (1.20–1.30)	<0.001
**Sex of household head**		
Male^Ref^	1.00	
Female	1.05 (0.99–1.11)	0.055
**Division**		
Barisal^Ref^	1.00	
Chittagong	1.36 (1.26–1.45)	<0.001
Dhaka	1.02 (0.95–1.10)	0.448
Khulna	0.95 (0.89–1.02)	0.202
Mymensingh	1.00 (0.93–1.08)	0.839
Rajshahi	0.99 (0.93–1.07)	0.994
Rangpur	1.06 (0.98–1.13)	0.105
Sylhet	1.30 (1.20–1.40)	<0.001
**Spousal age difference(Years)**		
≤5 (years) ^Ref^	1.00	
6 to 10 (years)	1.13 (1.09–1.18)	<0.001
≥11 years	1.20 (1.15–1.26)	<0.001
**Wealth Index**		
Poor^Ref^	1.00	
Middle	0.98 (0.93–1.03)	0.500
Rich	0.89 (0.85–0.94)	<0.001

**Key note:** ^Ref^Reference, ^HR^Hazard Ratio, ^CI^Confidence Interval, ^P-Value^Probability Value

Women whose age at first marriage was greater than or equal to 18 years had a 1.19 times higher hazard of giving first birth after marriage (HR: 1.19, 95% C.I = 1.15–1.24) than women aged less than 18 years. Women’s education and their partner’s education were positively associated with the first birth of women, and this association is significant with a 1% level of significance. The women who had primary education (HR: 1.13, 95% C.I = 1.07–1.19) and secondary and above education (HR: 1.07, 95% C.I = 1.01–1.14) had a higher hazard of having first birth compared to illiterate women. Women whose husbands had primary educated were 1.2 times more hazard of having a first birth (HR: 1.19, 95% C.I = 1.14–1.26) and secondary and higher education were 1.3 times more hazarded of having a first birth (HR: 1.31, 95% C.I = 1.25–1.37) compared to no educated person. Women aged 21 to 30 Years had a 2.5 times higher hazard of having a first birth (HR: 2.46, 95% C.I = 2.29–2.63), and women aged greater than 30 years had a 2.4 times greater hazard of having a first birth (HR: 2.36, 95% C.I = 2.20–2.53) compare to women aged less than 21 years old. Women who used anything to avoid getting pregnant had 2.4 times more hazards to having a first birth (HR: 2.43, 95% C.I = 2.25–2.62) than women who used nothing. Women’s contraceptive use status had a significant positive association with first childbirth. Women who used contraception had 1.3 times elevated hazard of having a first childbirth (HR: 1.25, 95% C.I = 1.20–1.30) than women who did’t use contraception. Women whose household heads were female had higher odds (HR: 1.05, 95% C.I = 0.99–1.11) of having a first birth than women who had the male household head. The spousal age difference was found to be a significant positive factor of FBI. Couples whose age difference was 6 to 10 years were 1.13 times more hazard (HR: 1.13, 95% C.I = 1.09–1.18) to have a first birth and whose age difference was greater than ten years were 1.2 times more hazard (HR: 1.20, 95% C.I = 1.15–1.26) to have first birth compared to those couples whose age differences was less than six years.

## Discussion

The main goal of this study was to compare the results of the Cox-PH model and the parametric accelerated failure time model (AFT) model to analyze the time to the first birth of ever-married women in Bangladesh and determine the best-fitted model. This study found women’s current age, age at first marriage, education, partner’s education, status of avoiding pregnancy, contraceptive use status, and spousal age difference as a significant predictor of the timing of first birth of ever married Bangladeshi women.

This study found the median time to first birth after women’s marriage to be 26 months, which is almost similar to the WHO-recommended median birth interval of greater than 2 years or 24 months [6] and the median age at first birth was 18 years. But another study of Bangladesh also observed a median time to first birth of 25 months for Bangladeshi women [54], which is different from this present study. A study in Jordan found the median time to first birth after marriage was 15 months [55], and an analysis of Ghana observed a median time to first birth of 24 months (two years) after marriage [43]. The results of these studies are parallel to the results of this study.

This study’s full model (Table 3) indicated approximately similar results in analyzing the impact of predictors for both the Cox-PH model and the three parametric AFT models. And after testing the goodness of fit test for the three parametric AFT models (Table 4), the Log-normal distribution had the lowest AIC as well as BIC value among all three parametric AFT models. So Log-normal model was considered as the best-fitted model for analyzing the data of the FBI. That means the three parametric AFT models (Weibull, Exponential, and Log-normal) outperformed over the common COX-PH model (Tables 2 and 3). This finding is consistent with the findings of previous studies [32, 33, 46, 56].

The results of the best-fitted model in Table 5 (Log-normal AFT model), in analyzing data of time to FBI, indicated that respondent’s age at first marriage (greater than equal to 18 years), respondent’s education (primary education and above secondary), partner’s education (primary education and above secondary), respondent’s age (21 to 30 years and greater than 30 years), used anything to avoid getting pregnant (yes), contraceptive use status (yes), sex of household head (female), and spousal age difference (6 to 10 years and greater than equal to 11 years) had a significant positive relationship with the FBI of Bangladeshi ever-married women. These results are consistent with previous studies’ findings [46, 57].

Results of this study showed that respondent’s age at first marriage had a significant negative influence on the FBI of women. Women who entered into married life before eighteen years old had a lower likelihood than those who entered into married life at age eighteen and above, indicating women who married at an older age (at age eighteen and above) had a shorter duration of FBI than those who marry at a young age (before eighteen years). This finding is similar to previous research [57].

Respondent’s education and their partner’s education had a negative relationship in analyzing FBI of Bangladeshi ever-married women. Women and their partners having primary, secondary, and above education had shorter FBI than the uneducated women. These findings are similar to the findings of earlier studies [18, 45, 55]. This might be because educated women and their partners spend a lot of time completing the educational part. As a result, they start their marital life lately. And may wish to give birth their first birth very quickly to cover the time of their late entry into married life, and they are financially stable enough to give birth to a child [39, 55].

Respondent’s current age was a significant factor that had a relation with FBI of ever married Bangladeshi women. Older women were more likely to have a short duration of giving their first birth, and the younger women were more likely to have a long birth interval. This is parallel to the previous study [58]. The reason behind this fact is the deducting nature of fertility with the increase of maternal age and different physiological disorders of different ages.

Contraceptive using status was identified as a significant predictor in analyzing FBI of ever-married Bangladeshi women. The birth interval was shorter among the women using any kind of contraceptive method than the women who didn’t use any method of contraception. These findings are unexpected, but it coincides with the previous studies [41, 42, 55, 59]. This might be the reason that women who didn’t use any method of contraception might have less education, lower age during the time of their first marriage, and might come from the countryside [41].

In this study, a higher gap between the age of women and their spouses was found to be associated with higher early motherhood of women, which is consistent with the previous studies [40, 44]. This might be a fact that a lower age gap can make a balanced marital relationship between husband and wife, allowing women to make self-decision on family welfare and family planning like contraceptives [60].

## Strength and limitation

The main strength of this study is that it was performed it’s analysis with a large sample size of a nationally representative secondary dataset Bangladesh Demographic and Health Survey (BDHS 2017–18). Most of the studies didn’t check the PH assumptions of the Cox-PH model and didn’t suggest any alternative process if the PH assumption is violated of that model. Analysis of this study encompassed several potential covariates which exhibited an association with FBI of Bangladeshi women. The models which have been used here for the alternative of the Cox-PH model are widely used analyzing methods. And it has no PH assumption like the Cox-PH model. Due to the unavailability and missing information of some related variables, we couldn’t include several potential covariates associated with the FBI of Bangladeshi women.

## Conclusion

In this study, we checked the PH assumption of the FBI according to different categorical variables. And we found a violation of the PH model for the FBI data of this study. We applied three commonly used AFT models (Weibull distribution, Exponential distribution, and Log-normal distribution). After comparing the goodness of fit of these three AFT models, we found log-normal distribution as the best-fitted model for this study. The results of best fitted log-normal AFT model exhibited that women’s age and age at first marriage, maternal and paternal education, contraceptive use status, used anything to avoid pregnancy, sex of household head, the spousal age difference had significant associations with FBI of ever married Bangladeshi women. Women who were educated, older, using a contraceptive method and having higher spousal age differences had shorter FBI. The Government of Bangladesh should take the necessary steps to make people more educated and conscious about contraceptive use. As higher age gap between women and their partners decreases the time of first birth of the child, there should have the least spousal age gap between women and their partners.

## Abbreviations

AFT: Accelerated Failure Time
AIC: Akaike Information Criterion
BDHS: Bangladesh Demographic and Health Survey
BIC: Bayesian Information Criterion
CI: Confidence Interval
Cox-PH: Cox Proportional Hazard
Df: Degree of Freedom
DHS: Demographic and Health Survey
EA: enumeration area
FBI: First Birth Interval
HR: Hazard Ratio
MOHFW: Ministry of Health and Family Welfare
NIPORT: National Institute of Population Research and Training
OR: Odds Ratio
P-Value: Probability Value
Ref: Reference
USAID: United States Agency for International Development

## References

[1] Gillespie DO, Russell AF, Lummaa V. The effect of maternal age and reproductive history on offspring survival and lifetime reproduction in preindustrial humans. Evolution. 2013;67(7):1964–74. doi: 10.1111/evo.12078 23815653

[2] Kamal A, Pervaiz MK. Determinants of marriage to first birth interval in Pakistan. Journal of Statistics. 2013;20(1).

[3] Kozuki N, Lee AC, Silveira MF, Victora CG, Adair L, Humphrey J, et al. The associations of birth intervals with small-for-gestational-age, preterm, and neonatal and infant mortality: a meta-analysis. BMC public health. 2013;13(3):1–9. doi: 10.1186/1471-2458-13-S3-S3 24564484 PMC3847557

[4] Nisha MK, Alam A, Islam MT, Huda T, Raynes-Greenow C. Risk of adverse pregnancy outcomes associated with short and long birth intervals in Bangladesh: evidence from six Bangladesh Demographic and Health Surveys, 1996–2014. BMJ open. 2019;9(2):e024392. doi: 10.1136/bmjopen-2018-024392 30798311 PMC6398728

[5] Miller R, Karra M. Birth spacing and child health trajectories. Population and Development Review. 2020;46(2):347–71.

[6] Aleni M, Mbalinda S, Muhindo R. Birth intervals and associated factors among women attending young child clinic in Yumbe Hospital, Uganda. International journal of reproductive medicine. 2020;2020. doi: 10.1155/2020/1326596 31984212 PMC6964709

[7] Conde‐Agudelo A, Rosas‐Bermudez A, Castaño F, Norton MH. Effects of birth spacing on maternal, perinatal, infant, and child health: a systematic review of causal mechanisms. Studies in family planning. 2012;43(2):93–114. doi: 10.1111/j.1728-4465.2012.00308.x 23175949

[8] Cleland J, Conde-Agudelo A, Peterson H, Ross J, Tsui A. Contraception and health. The Lancet. 2012;380(9837):149–56. doi: 10.1016/S0140-6736(12)60609-6 22784533

[9] National Institute of Population Research and Training, ICF. *Bangladesh Demographic and Health Survey 2017–18*: *Key Indicators*. Dhaka, Bangladesh, and Rockville, Maryland, USA: NIPORT, and ICF, 2019.

[10] Rabbi AMF, Karmaker SC, Mallick SA, Sharmin S. Determinants of birth spacing and effect of birth spacing on fertility in Bangladesh. Dhaka University Journal of Science. 2013;61(1):105–10.

[11] Dibaba Y. Child spacing and fertility planning behavior among women in mana district, Jimma Zone, South West Ethiopia. Ethiopian journal of health sciences. 2010;20(2). doi: 10.4314/ejhs.v20i2.69433 22434965 PMC3275838

[12] Fotso JC, Cleland J, Mberu B, Mutua M, Elungata P. Birth spacing and child mortality: an analysis of prospective data from the Nairobi urban health and demographic surveillance system. Journal of biosocial science. 2013;45(6):779–98. doi: 10.1017/S0021932012000570 22958417 PMC3785173

[13] Rahman MS, Howlader T, Masud MS, Rahman ML. Association of low-birth weight with malnutrition in children under five years in Bangladesh: do mother’s education, socio-economic status, and birth interval matter? PloS one. 2016;11(6):e0157814. doi: 10.1371/journal.pone.0157814 27355682 PMC4927179

[14] Alam MM. Marriage to first birth interval and its associated factors in Bangladesh. Asian Journal of Social Sciences & Humanities. 2015;4(4):36–47.

[15] Singh N, Narendra R, Hemochandra L. Determinants of waiting time to conception in Manipuri women. Kuwait Medical Journal. 2007;39(1):39–43.

[16] Purnami SW, Aida FN, Sutikno S, Herowati D, Sjafii A, Wibisono SP, et al. Survival analysis to determine age to give first birth in women in east Java using extended Cox regression. Jurnal Biometrika dan Kependudukan. 2021;10(2):144–52.

[17] Sahara N, Idris I, Putri DZ. Faktor-faktor yang mempengaruhi keputusan wanita menikah di Sumatera Barat. Jurnal Ecogen. 2019;1(3):640–7.

[18] Gurmu E, Etana D. Age at first marriage and first birth interval in Ethiopia: analysis of the roles of social and demographic factors. African Population Studies. 2014;28(3):1332–44.

[19] Islam S. Differential determinants of birth spacing since marriage to first live birth in rural Bangladesh. Pertanika J Soc Sci Hum. 2009;17(1):1–6.

[20] Fagbamigbe AF, Idemudia ES. Survival analysis and prognostic factors of timing of first childbirth among women in Nigeria. BMC pregnancy and childbirth. 2016;16:1–12. 27178189 10.1186/s12884-016-0895-y PMC4867998

[21] DaVanzo J, Hale L, Razzaque A, Rahman M. Effects of interpregnancy interval and outcome of the preceding pregnancy on pregnancy outcomes in Matlab, Bangladesh. BJOG: An International Journal of Obstetrics & Gynaecology. 2007;114(9):1079–87. doi: 10.1111/j.1471-0528.2007.01338.x 17617195 PMC2366022

[22] Saha UR, van Soest A. Infant death clustering in families: magnitude, causes, and the influence of better health services, Bangladesh 1982–2005. Population studies. 2011;65(3):273–87. doi: 10.1080/00324728.2011.602100 21916660

[23] Casterline JB, Odden C. Trends in inter-birth intervals in developing countries 1965–2014. Population and Development Review. 2016:173–94.

[24] De Jonge HC, Azad K, Seward N, Kuddus A, Shaha S, Beard J, et al. Determinants and consequences of short birth interval in rural Bangladesh: a cross-sectional study. BMC pregnancy and childbirth. 2014;14(1):1–7. doi: 10.1186/s12884-014-0427-6 25539669 PMC4314752

[25] Exavery A, Mrema S, Shamte A, Bietsch K, Mosha D, Mbaruku G, et al. Levels and correlates of non-adherence to WHO recommended inter-birth intervals in Rufiji, Tanzania. BMC pregnancy and childbirth. 2012;12(1):1–8. doi: 10.1186/1471-2393-12-152 23237623 PMC3573999

[26] Khan JR, Bari W, Latif A. Trend of determinants of birth interval dynamics in Bangladesh. BMC public health. 2016;16(1):1–11. 27595598 10.1186/s12889-016-3577-9 PMC5011889

[27] Mitra SN, M. Nawab Ali, Shahidul Islam, Anne R. Cross, and Tulshi Saha. Bangladesh Demographic and Health Survey 1993–1994. Calverton, Maryland, USA: National Institute of Population Research and Training—NIPORT/Bangladesh, Mitra and Associates/Bangladesh, and Macro International, 1994.

[28] Rabbi AMF, Kabir M. Factors influencing age at first birth of Bangladeshi women-A multivariate approach. American Journal of Public Health Research. 2013;1(7):191–5.

[29] Rutstein SO. Effects of preceding birth intervals on neonatal, infant and under‐five years mortality and nutritional status in developing countries: evidence from the demographic and health surveys. International Journal of Gynecology & Obstetrics. 2005;89:S7–S24. doi: 10.1016/j.ijgo.2004.11.012 15820369

[30] Giviziez CR, Sanchez EG, Approbato MS, Maia MC, Fleury EAB, Sasaki RS. Obesity and anovulatory infertility: a review. JBRA assisted reproduction. 2016;20(4):240. doi: 10.5935/1518-0557.20160046 28050960 PMC5265624

[31] Mahmood S, Zainab B, Latif AM. Frailty modeling for clustered survival data: an application to birth interval in Bangladesh. Journal of Applied Statistics. 2013;40(12):2670–80.

[32] Teshnizi SH, Ayatollahi SMT. Comparison of cox regression and parametric models: Application for assessment of survival of pediatric cases of acute leukemia in Southern Iran. Asian Pacific journal of cancer prevention: APJCP. 2017;18(4):981. 28545196 10.22034/APJCP.2017.18.4.981 PMC5494248

[33] Adelian R, Jamali J, Zare N, Ayatollahi S, Pooladfar G, Roustaei N. Comparison of Cox’s regression model and parametric models in evaluating the prognostic factors for survival after liver transplantation in Shiraz during 2000–2012. International journal of organ transplantation medicine. 2015;6(3):119. 26306158 PMC4545306

[34] Ware JE Jr, Kosinski M, Keller SD. A 12-Item Short-Form Health Survey: construction of scales and preliminary tests of reliability and validity. Medical care. 1996:220–33. doi: 10.1097/00005650-199603000-00003 8628042

[35] Khanal SP, Sreenivas V, Acharya SK. Accelerated failure time models: an application in the survival of acute liver failure patients in India. Int J Sci Res. 2014;3(6):161–6.

[36] Ahammed B, Kabir MR, Abedin MM, Ali M, Islam MA. Determinants of different birth intervals of ever married women: Evidence from Bangladesh. Clinical Epidemiology and Global Health. 2019;7(3):450–6.

[37] Bangladesh: Standard DHS, 2017–18 Dataset [Internet]. 2020 [cited 2021]. https://dhsprogram.com/data/dataset/Bangladesh_Standard-DHS_2017.cfm?flag=1.

[38] Khan PMK. Birth weight and growth of our babies—where are we? The Daily Star. 2017.

[39] Chernet AG, Shebeshi DS, Banbeta A. Determinant of time-to-first birth interval after marriage among Ethiopian women. BMC Women’s Health. 2019;19(1):1–6. 31822276 10.1186/s12905-019-0858-3 PMC6905102

[40] Karim MA, Faruk MO. Patterns and Differentials of Age at First Motherhood Among Married Adolescents in Bangladesh. Biomedical Sciences. 2021;7(3):86.

[41] Chowdhury AH, Karim A. Patterns and differentials of birth intervals in Bangladesh. Global Journal of Science Frontier Research Interdisciplinar. 2013;13(2).

[42] Hidayat R, Sumarno H, Nugrahani EH. Survival analysis in modeling the birth interval of the first child in Indonesia. Open Journal of Statistics. 2014;4(03):198.

[43] Logubayom IA, Luguterah A. Survival analysis of time to first birth after marriage. Survival. 2013;3(12).

[44] Yimer R, Mekonnen F, Seretew W. Time to first birth and its Predictors among reproductive-age women in Ethiopia: inverse Weibull gamma shared frailty model. 2020. 10.1186/s12905-021-01254-z PMC7980321 33740957

[45] Marphatia AA, Ambale GS, Reid AM. Women’s Marriage Age Matters for Public Health: A Review of the Broader Health and Social Implications in South Asia. Frontiers in Public Health. 2017;5. doi: 10.3389/fpubh.2017.00269 29094035 PMC5651255

[46] Faruk A, editor The comparison of proportional hazards and accelerated failure time models in analyzing the first birth interval survival data. Journal of Physics: Conference Series; 2018: IOP Publishing.

[47] Shayan Z, Ayatollahi SMT, Zare N, Moradi F. Prognostic factors of first birth interval using the parametric survival models. Iranian journal of reproductive medicine. 2014;12(2):125. 24799870 PMC4009565

[48] Harrell J, Frank E, Harrell FE. Cox proportional hazards regression model. Regression modeling strategies: With applications to linear models, logistic and ordinal regression, and survival analysis. 2015:475–519.

[49] Nadarajah S, Haghighi F. An extension of the exponential distribution. Statistics. 2011;45(6):543–58.

[50] Lahcene B. Extended lognormal distribution: Properties and applications. World Scientific News. 2020;145:16–30.

[51] Lai C, Murthy D, Xie M. Weibull distributions. Wiley Interdisciplinary Reviews: Computational Statistics. 2011;3(3):282–7.

[52] Bozdogan H. Akaike’s information criterion and recent developments in information complexity. Journal of mathematical psychology. 2000;44(1):62–91. doi: 10.1006/jmps.1999.1277 10733858

[53] Zucchini W. An Introduction to Model Selection. J Math Psychol. 2000;44(1):41–61. Epub 2000/03/29. doi: 10.1006/jmps.1999.1276 . 10733857

[54] Rahman M, Mustafi M, Azad M. Analysis of the determinant’s of marriage to first birth interval in Bangladesh. International Journal of Management and Sustainability. 2013;2(12):208–19.

[55] Al Shanfari NSS, Islam MM. Survival Analysis and Predictors of Time to First Birth After Marriage in Jordan.

[56] Kargarian-Marvasti S, Rimaz S, Abolghasemi J, Heydari I. Comparing of Cox model and parametric models in analysis of effective factors on event time of neuropathy in patients with type 2 diabetes. Journal of research in medical sciences: the official journal of Isfahan University of Medical Sciences. 2017;22. doi: 10.4103/jrms.JRMS_6_17 29184573 PMC5680655

[57] Gebeyehu A. Survival analysis of time-to-first birth after marriage among women in Ethiopia: application of parametric shared frailty model. Department of Statistics, College of Natural Sciences, Jimma University, Jimma. 2015.

[58] Fallahzadeh H, Farajpour Z, Emam Z. Duration and determinants of birth interval in Yazd, Iran: a population study. Iranian journal of reproductive medicine. 2013;11(5):379. 24639769 PMC3941411

[59] Sathar Z. Birth spacing in Pakistan. Journal of biosocial science. 1988;20(2):175–94. doi: 10.1017/s0021932000017417 3384834

[60] Unicef. Early marriage: child spouses. 2001.

